# 

**Published:** 1873-05

**Authors:** 


### PUBLISHERS' NOTICE.

*Our* NEW CATALOGUE *of Medical and Surgical Works*

IS MAILED THIS MONTH TO EVERY SUBSCRIBER TO THE JOURNAL, WITH THE PRICES ANNEXED AT WHICH THEY WILL BE SENT BY MAIL, FREE OF POSTAGE, UPON RECEIPT OF THE MONEY.

### HOW TO PROCURE BOOKS.

We will forward per U. S. Mail, and prepay postage upon the same, all books named in this Catalogue, weighing four pounds and under, (Books weighing over four pounds are not mailable,) upon receipt of Publishers' Prices, as named herein.

☞ We assume no risk upon Money or Books, although out of the many hundreds of volumes sent by us by mail, none to our knowledge have been lost.

We will forward Books to all points reached by the REGULAR EXPRESS LINES, east of the Mississippi River, and prepay charges on the same, upon the receipt of Publishers' Prices. Money to accompany all orders.

☞ When Books are forwarded per EXPRESS "COLLECT ON DELIVERY," all expenses of freight and of returning the money, must be paid by the consignee.

The safest mode of remittance is by bank check, or postal money order, drawn to our order. Where these are not accessible, remittances may be made at our risk, by forwarding in registered letters.

Address—

W. B. KEEN, COOKE & CO.,

113 & 115 *State Street, Chicago.*

### *The Illinois State Medical Society.*

The Twenty-third Annual Session of this Society, will be held in Bloomington, Illinois, May 20th, 1873, at 10 A.M.

The following Committees are expected to report.

STANDING COMMITTEES.

*Practical Medicine.*—S. K. Crawford, M.D., Monmouth ; D. L. Jewett, M.D., Watseka ; H. M. Lyman, M.D., Chicago.

*Surgery.*—J. L. White, M.D., Bloomington ; J. B. Hamilton, M.D., Kane ; H. W. Kendall, M.D., Quincy.

*Ophthalmology.*—E. L. Holmes, M.D., Chicago ; J. P. Johnson, M.D., Peoria ; F. C. Hotz, M.D., Chicago.

*Otology.*—S. J. Jones, M.D., Chicago ; Chas. Hunt, M.D., Dixon ; Thos. Galt, M.D., Rock Island.

*Obstetrics.*—T. D. Fitch, M.D., Chicago ; A. Niles, M.D., Quincy ; D. S. Jenks, M.D., Plano.

*Drugs and Medicines.*—J. H. Hollister, M.D., Chicago ; E. P. Cook, M.D., Mendota ; J. L. Hamilton, M.D., Peoria.

*Necrology.*—G. W. Albin, M.D., Neoga ; J. O. Hamilton, M.D., Jerseyville ; J. K. Secord, M.D., Elmwood.

SPECIAL COMMITTEES.

*Galvano-Therap.*—D. Prince, M.D., Jacksonville.
*Hernia.*—J. H. Reeder, M.D., Lacon.
*Hygiene.*—T. F. Worrell, M.D., Bloomington.
*On the Assistance Necessary and Justifiable in Difficult and Protracted Labors.*—S. B. Hawley, M.D., Aurora.
*Stricture of the Urethra.*—John Murphy, M.D., Peoria.
*Phthisis Pulmonalis.*—H. A. Johnson, M.D., Chicago.
*Phys. Nerv. Syst.*—C. W. Earl, M.D., Chicago.
*Cerebro-Spinal Meningitis.*—G. Wheeler Jones, Danville.
*Locomotor Ataxia.*—N. S. Davis, M.D., Chicago.
*Orthopedic Surgery.*—J. S. Sherman, M.D., Chicago.
*To Prepare and Deliver a Public Address at the next Meeting of the Society.*—George T. Allen, M.D., Springfield.

